# Orolabial Lymphogranuloma Venereum, Michigan, USA 

**DOI:** 10.3201/eid2511.190819

**Published:** 2019-11

**Authors:** Sahrish Ilyas, Deborah Richmond, Gerald Burns, Katherine E. Bowden, Kimberly Workowski, Ellen N. Kersh, Pranatharthi H. Chandrasekar

**Affiliations:** Wayne State University School of Medicine, Detroit, Michigan, USA (S. Ilyas, D. Richmond, G. Burns, P.H. Chandrasekar);; Centers for Disease Control and Prevention, Atlanta, Georgia, USA (K.E. Bowden, K. Workowski, E.N. Kersh);; Emory University, Atlanta (K. Workowski)

**Keywords:** Lymphogranuloma venereum, men who have sex with men, MSM, orolabial site, cervical adenopathy, Chlamydia trachomatis, bacteria, Michigan, USA

## Abstract

Orolabial lymphogranuloma venereum was diagnosed for a man in Michigan, USA, who had sex with men, some infected with HIV. High index of suspicion for lymphogranuloma venereum led to accurate diagnosis, successful therapy, and description of an L2b variant with a unique genetic mutation.

Lymphogranuloma venereum (LGV) is typically a genital ulcer disease with inguinal adenopathy, caused by L serovars of *Chlamydia trachomatis*. Most commonly today, the infection causes proctocolitis, particularly in men who have sex with men (MSM) with HIV infection. We describe a case of LGV at an unusual site (orolabial) with submandibular adenopathy in a man with a history of having sex with men with advanced HIV infection.

## The Study

In February 2019, a 25-year-old man with a history of having sex with men with advanced HIV infection sought care at the Wayne State University Physician Group Infectious Diseases Clinic in Detroit, Michigan, USA, for 2 large ulcers over his lower lip. He had noticed the ulcers 2 weeks earlier, associated with pain and purulent discharge, along with a rapidly enlarging, painful swelling over his upper left neck. He reported no history of fever or chills. He also reported having had unprotected insertive and receptive oral and anal sex with >30 male partners during the previous year. He reported that he found anonymous sexual contacts by using “sex apps” and did not trade sex for drugs or money. In 2007, the patient had received a diagnosis of HIV infection and was taking antiretroviral drugs intermittently. His medical history included syphilis, gonorrhea, and genital chlamydia infections.

Physical examination revealed that the patient was afebrile with stable vital signs. His lower lip was markedly swollen and tender, with 2 large, purulent ulcers: 1 deep ulcer on the central lower lip and 1 over the left side near the angle of his mouth. Also, left-sided submandibular adenopathy was present as a large (≈10 cm diameter), tender, nonfluctuant swelling, which was neither warm nor erythematous. His oropharynx, buccal mucosa, and tongue appeared normal. Results of the rest of the examination, including the external genitalia, groin, and perianal region, were unremarkable.

Diagnostic testing of the lip ulcer included nucleic acid amplification testing (NAAT) for herpes simplex viruses (HSVs) 1 and 2 and *Chlamydia* (Roche LightCycler real-time PCR for HSVs 1 and 2, https://diagnostics.roche.com; BD Viper XTR technology for GC/CT NAAT, https://www.bd.com). We performed NAAT on pharyngeal samples to test for *C. trachomatis* and *Neisseria gonorrhoeae*. Additional diagnostics included rapid plasma reagin testing, serologic testing for treponema, HIV viral load measurement, and CD4+ lymphocyte count. Valacyclovir was empirically prescribed; however, the patient did not fill this prescription. 

*C. trachomatis* was identified in samples from the lip and throat; NAAT results for HSVs and *N. gonorrheae* were negative. The patient’s HIV load was 300,000 copies/mL, and CD4+ lymphocyte count was 73/µL. Oral doxycycline (100 mg 2×/d for 3 weeks) was prescribed, and the lip ulcers and cervical adenopathy resolved over the next month. Concomitantly, appropriate antiretroviral therapy was initiated. 

Swab samples of the lip ulcers were submitted to the San Francisco Public Health Department, where diagnosis of LGV was confirmed by use of a laboratory-developed, Clinical Laboratory Improvement Amendments–approved test (*1*). The lip ulcer samples then underwent LGV genotyping by sequencing the entire *ompA* gene at the Centers for Disease Control and Prevention (CDC). LGV genotyping confirmed *C. trachomatis* serovar L2b on the basis of sequence variations compared with serovar L2 (*2*). Furthermore, the *ompA* gene, which encodes the chlamydial major outer membrane protein (MOMP), contained a 5′ deletion of 1–82 nt in constant domain (CD) 1 and a 3′ deletion of 904–1,185 nt, spanning CD4, variable domain (VD) 4, and CD5. 

## Conclusions

The prompt diagnosis of LGV infection involving the lip with resultant submandibular adenopathy in this patient was based on a high index of clinical suspicion and specialized laboratory testing. Additional testing at CDC confirmed *C. trachomatis* serovar L2b. 

Rare cases of oral/oropharyngeal LGV have been described (Table) (*3**–**9*). For patients who engage in high-risk sexual behavior, with anal–oral contact, LGV is a possible cause of orolabial/oropharyngeal infection. The substantial cervical lymphadenopathy in this patient was reminiscent of inguinal bubo associated with genital LGV, which may serve as a diagnostic clue. Moreover, clinical manifestations in the ongoing epidemic of LGV among MSM show a shift from the classical inguinal form (inguinal adenopathy with penile lesion) to the anorectal form. Our report of orolabial LGV adds to the possibility of gastrointestinal tract infection in this epidemic, perhaps explaining the discrepancy in the ratio of anorectal LGV to inguinal LGV cases (*10*).

**Table:** Reported cases of oropharyngeal LGV worldwide*

Year	Country	Specimen site	Genotype†	Prevalence of CT+ oropharyngeal samples	Reference
1974	Thailand	Oral ulcer	LGV	Single patient	(*3*)
2011	United Kingdom	Throat	LGV	1/51 (2%)	(*4*)
2012	Finland	Pharynx	L2b	1/56 (2%)	(*5*)
2013	United Kingdom	Oral ulcer	LGV	4/4 (100%)	* (* *6* *)*
2015	Netherlands	Pharynx	LGV	2/23 (8.7%)	(*7*)
2018	France	Oropharynx	L2, L2b	6/62 (9.7%)	(*8*)
2019	Spain	Tongue ulcer	LGV	Single patient	(*9*)

*CT+, *Chlamydia trachomatis*–positive; LGV, lymphogranuloma venereum. †L2 and L2b variant genotypes were determined by sequence variations in *ompA* (*2*). LGV genotypes were determined according to the methods described in each study, either anti-LGV complement fixation test or LGV reverse transcription PCR.

In the past several years, reports of LGV have been increasing in western Europe and the United States, primarily among MSM (*11*). An outbreak of 38 cases of LGV during August 2015–April 2016 encountered at the Wayne State University Physician Group Infectious Diseases Clinic in Detroit was recently reported and occurred among MSM with HIV infection (*12*). Among the 38 cases, 21 (55%) were confirmed by the CDC laboratory–developed LGV NAAT (*2*) on the basis of 19 positive rectal swab specimens and 2 positive swab samples from penile lesions; treatment with doxycycline (100 mg 2×/d for 21 days) was successful for all patients. Although the L2b serovar was correlated with a proctitis outbreak among MSM in Amsterdam in 2000, no cases of LGV at the orolabial site were detected in this outbreak or a 1980s outbreak in San Francisco, California, USA (*13*).

The identification of 5′ and 3′ deletions in *ompA* indicate a novel subtype of serovar L2b, further highlighting the rarity of this patient’s case. Chlamydial MOMP functions as a porin with VD1, VD2, and VD4 at the surface of the chlamydial elementary body directed toward the external environment and host cells. VD4 encodes subspecies-specific neutralizing epitopes (*14*,*15*). It remains unclear how or whether the deletions in CD1, CD4, VD4, and CD5 characterized in this case affect pathogenicity and tissue tropism. Identification of rare clinical manifestations such as this warrant further genetic investigation to provide more information about the molecular mechanisms behind the pathogenesis and transmission of chlamydia.

A widely available Food and Drug Administration–approved molecular test for diagnosis of LGV would be highly useful, especially at the point of care. In its absence, a probable LGV case can be supported by *C. trachomatis* NAAT positivity of lesion specimens. However, extragenital specimens (e.g., from a lip lesion) are currently not approved by the Food and Drug Administration as a specimen type for commercial *C. trachomatis* NAAT, which poses a challenge for laboratories because they must perform validations for such specimens for their Clinical Laboratory Improvement Amendments certification. Similar obstacles apply to laboratory-developed LGV-specific testing. As a result, the availability of specific diagnostic testing is scarce. At present, to ensure prompt resolution of symptoms, prevention of complications, and treatment of the sex partner, all suspected cases should be presumptively treated while awaiting diagnostic evaluation.

In summary, this case indicates that LGV infection should be considered for patients, especially MSM, with orolabial lesions and cervical adenopathy. Successful treatment of the patient reported here was based on a *C. trachomatis*–positive NAAT result, and LGV specialized testing served as a supplement for full investigation of this unusual case.
